# Nitric Oxide Released from Luminal S-Nitroso-N-Acetylcysteine Increases Gastric Mucosal Blood Flow

**DOI:** 10.3390/molecules20034109

**Published:** 2015-03-04

**Authors:** Gabriela F. P. de Souza, Patricia Taladriz-Blanco, Lício A. Velloso, Marcelo G. de Oliveira

**Affiliations:** 1 Institute of Chemistry, University of Campinas, UNICAMP, CP 6154, Campinas, SP 13083-970, Brazil; E-Mails: hbee_quimica@yahoo.com.br (G.F.P.S.); patricia.taladriz@iqm.unicamp.br (P.T.-B.); 2 Faculty of Medical Sciences, University of Campinas, UNICAMP, CP, Campinas, SP 13084-970, Brazil; E-Mail: lavelloso.unicamp@gmail.com

**Keywords:** nitric oxide, S-nitroso-N-acetylcysteine, gastric blood flow, vasodilation

## Abstract

Nitric oxide (NO)-mediated vasodilation plays a key role in gastric mucosal defense, and NO-donor drugs may protect against diseases associated with gastric mucosal blood flow (GMBF) deficiencies. In this study, we used the *ex vivo* gastric chamber method and Laser Doppler Flowmetry to characterize the effects of luminal aqueous NO-donor drug S-nitroso-N-acetylcysteine (SNAC) solution administration compared to aqueous NaNO_2_ and NaNO_3_ solutions (pH 7.4) on GMBF in Sprague-Dawley rats. SNAC solutions (600 μM and 12 mM) led to a rapid threefold increase in GMBF, which was maintained during the incubation of the solutions with the gastric mucosa, while NaNO_2_ or NaNO_3_ solutions (12 mM) did not affect GMBF. SNAC solutions (600 μM and 12 mM) spontaneously released NO at 37 °C at a constant rate of 0.3 or 14 nmol·mL^−1^·min^−1^, respectively, while NaNO_2_ (12 mM) released NO at a rate of 0.06 nmol·mL^−1^·min^−1^ and NaNO_3_ (12 mM) did not release NO. These results suggest that the SNAC-induced GMBF increase is due to their higher rates of spontaneous NO release compared to equimolar NaNO_2_ solutions. Taken together, our data indicate that oral SNAC administration is a potential approach for gastric acid-peptic disorder prevention and treatment.

## 1. Introduction

In mammalian cells nitric oxide (NO) is synthesized by NO synthases (NOS) from L-arginine, and it mediates several biological processes, including neurotransmission, vasodilation, host defense and immunity [1]. NO-mediated vasodilation is a fundamental gastric mucosa defense mechanism, as impaired blood supply to the stomach renders the mucosa more susceptible to acid and pepsin-induced injuries [2]. In addition, the NO actions on gastric physiology include the modulation of gastric mucous formation [3] and antimicrobial activity [4], the reduction of mast cell degranulation and release, neutrophil adherence and secretion, macrophage cytokine release and epithelial barrier function regulation [5,6,7] and its possible involvement in the protective effects of angiotensin (1–7) on gastroesophageal reflux [8].

Cyclooxygenase inhibition in the gastrointestinal mucosa by nonsteroidal anti-inflammatory drugs (NSAIDs) is associated with GMBF alterations, which leads to gastrointestinal injury as a common NSAID side effect [9]. Widespread and intense NSAID use has generated an important demand for gastrointestinal injury prevention, which has led to modified NSAID design and development in which NO donor moieties are chemically attached to allow for local NO release in the stomach. This gastric mucosal blood flow regulation by combining NSAIDs with local NO release proved to be a valid and efficacious approach to prevent NSAID-induced gastric lesions [10]. As new modified NSAID development requires novel synthetic routes and clinical trials, an alternative approach is the development of formulations, which combine standard oral NSAIDs with an NO donor drug in a physical mixture. The efficacy of this combination was recently reported by Tam *et al.* [11], who showed that co-administration of the well-known NO donor S-nitrosoglutathione (GSNO) with piroxicam inhibited NSAID-associated gastric lesions.

**Figure 1:**
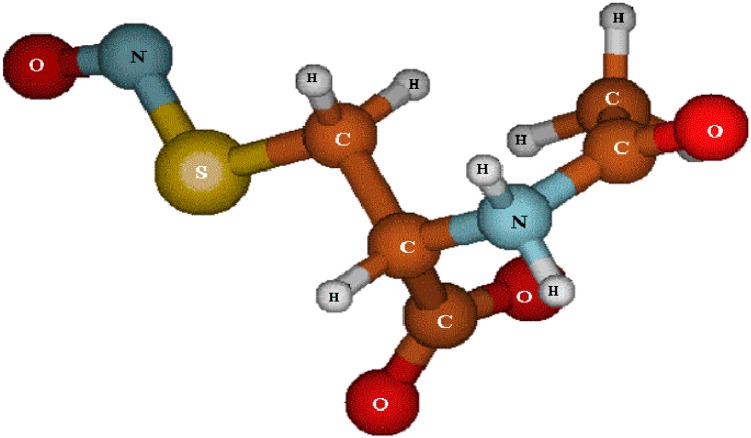
Structure of S-nitroso-N-acetylcysteine (SNAC).

Like GSNO, S-nitroso-N-acetylcysteine (SNAC) (Figure 1) is a primary S-nitrosothiol (RSNO) derived from N-acetylcysteine (NAC) S-nitrosation. Both GSNO and SNAC have been shown to be potent vasodilators *in vivo* [12,13], and oral aqueous SNAC administration in animals with impaired NO production have therapeutic effects [14,15,16]. However, SNAC’s vasodilator effect on GMBF and its potential for protecting the gastric mucosa against acids or pepsin injuries has not been investigated.

In this study, we evaluated the effect of luminal SNAC solution administration on Sprague Dawley rat GMBF compared to the effects of sodium nitrite (NaNO_2_) and sodium nitrate (NaNO_3_) solutions at pH 7.4 using Laser Doppler Flowmetry. We observed a significant increase in GMBF upon SNAC solution treatment, but NaNO_2_ and NaNO_3_ solutions had no effect. The GMBF increase correlated with the spontaneous NO release from SNAC, as measured by chemiluminescence.

## 2. Results and Discussion

### 2.1. NO Release Profiles

**Figure 2:**
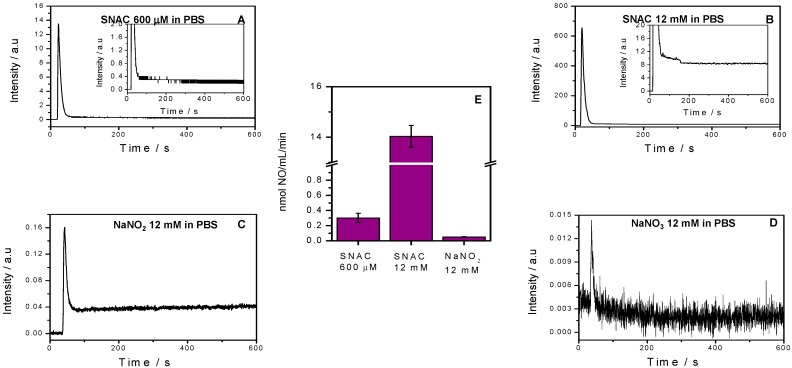
Chemiluminescence detection of NO release from 12 mM SNAC (**A**) and 600 μM SNAC (**B**) and 12 mM NaNO_2_ and NaNO_3_ solutions (**C** and **D**, respectively) over 10 min at 37 °C, and the total NO release (**E**) extracted from these curves. The baseline trace was recorded for PBS, pH 7.4. Error bars are the SEM of triplicates. a.u., arbitrary units.

Figure 2 shows the chemiluminescence profiles of spontaneous NO released from 600 μM and 12 mM SNAC (A, B) and from 12 mM NaNO_2_ and NaNO_3_ solutions (C, D) over 10 min at 37 °C and pH 7.4, along with the corresponding total released NO, which was calculated from these curves (E). We observed a narrow chemiluminescence peak in each curve immediately after solution injection. After these initial peaks, NO release rates for the 600 µM SNAC, 12 mM SNAC and 12 mM NaNO_2_ plateaued above the initial baseline, and maintained their level over the remaining measurement times. The shift of these plateaus from the baseline can be seen more clearly in the insets of Figure 2A,B. After NaNO_3_ injection, the chemiluminescence signal did not shift from the baseline, and the extremely narrow initial peak was too low to quantify. We obtained total released NO values after SNAC and NaNO_2_ injection (expressed per mL per min) from the integrations of total area under the curves (over 600 s) (Figure 2E). The NO release rates from 600 μM and 12 mM SNAC were 0.3 nmol·mL^−1^·min^−1^ and 14 nmol·mL^−1^·min^−1^, respectively, compared to 0.06 nmol·mL^−1^·min^−1^ for the NaNO_2_ solution. Therefore, 12 mM SNAC releases NO at a rate 46 times higher than 600 μM SNAC, and 600 μM SNAC and 12 mM SNAC release NO at rates 5 and 233 times higher than 12 mM NaNO_2_ solution, respectively.

### 2.2. Gastric Blood Flow Measurements by Laser Doppler Flowmetry

**Figure 3:**
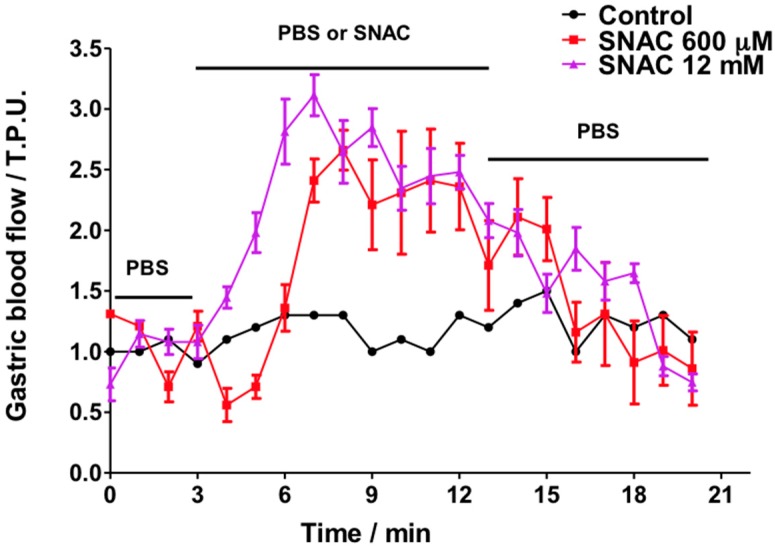
*Ex vivo* Laser Doppler measurements of rat gastric mucosal blood flow in a gastric chamber after incubation with PBS only over 20 min (control) or PBS for 3 min, followed by 600 μM or 12 mM SNAC for the next 10 min, which were replaced by PBS during the last 7 min, as indicated by the horizontal bars. The results are expressed as the mean ± S.E.M. of triplicates.

**Figure 4:**
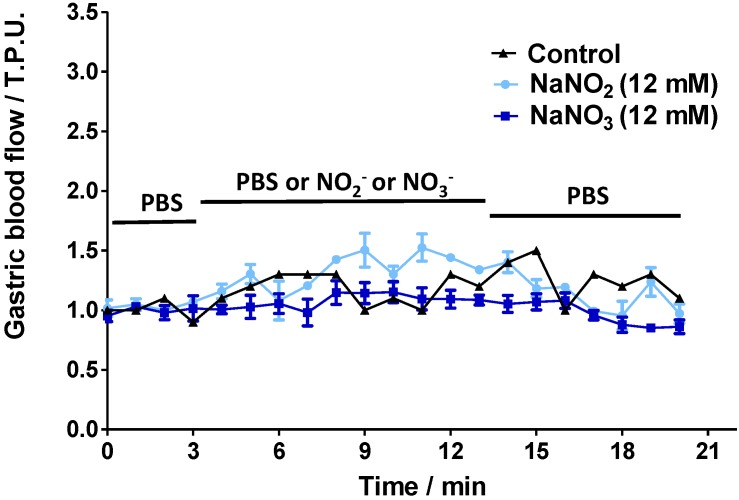
*Ex vivo* Laser Doppler measurements of rat gastric mucosal blood flow in a gastric chamber after incubation with PBS only over 20 min (control) or PBS solution for 3 min, followed by 12 mM NaNO_2_ or NaNO_3_ solutions for the next 10 min, which were replaced by PBS during the last 7 min, as indicated by the horizontal bars. The results are expressed as the mean ± S.E.M. of triplicates.

Figure 3 shows the GMBF profiles for control and SNAC-treated animals. Importantly, control animals were exposed only to PBS solution for the full 20 min (basal readings plus test reading), while the gastric mucosas of the treated animals were incubated in PBS during the first 3 min to record their basal GMBF, then the PBS was replaced by SNAC, NaNO_2_ or NaNO_3_. The GMBF of the control group remained stable throughout the 20-min period at a value of approximately 1.0 tissue perfusion units (T.P.U). The treated groups had similar stable baseline GMBF values in PBS during the first 3 min. However, when the PBS was replaced by SNAC, the GMBF rapidly increased with onsets at 3 and 5 min after 12 mM and 600 μM SNAC application, respectively. Both SNAC treatment groups reached similar GMBF peaks (2.5–3 T.P.U) after 4 to 5 min. In addition, increased GMBF values remained close to the peak values during SNAC incubation periods (from 3 to 13 min), but they slowly decreased after SNAC was replaced with PBS over the next 7 min until reaching the basal level after approximately 4 min. Notably, the 12 mM SNAC solution, which has a concentration 20 times higher than the 600 μM solution, led only to a slightly earlier increase in GMBF onset but not to a higher peak or a slower return to the baseline. In contrast to the GMBF profiles after SNAC application, luminal application of 12 mM NaNO_2_ or NaNO_3_ did not significantly change the basal GMBF over the same time periods (Figure 4).

### 2.3. Quantification of Plasma NOx Levels

**Figure 5:**
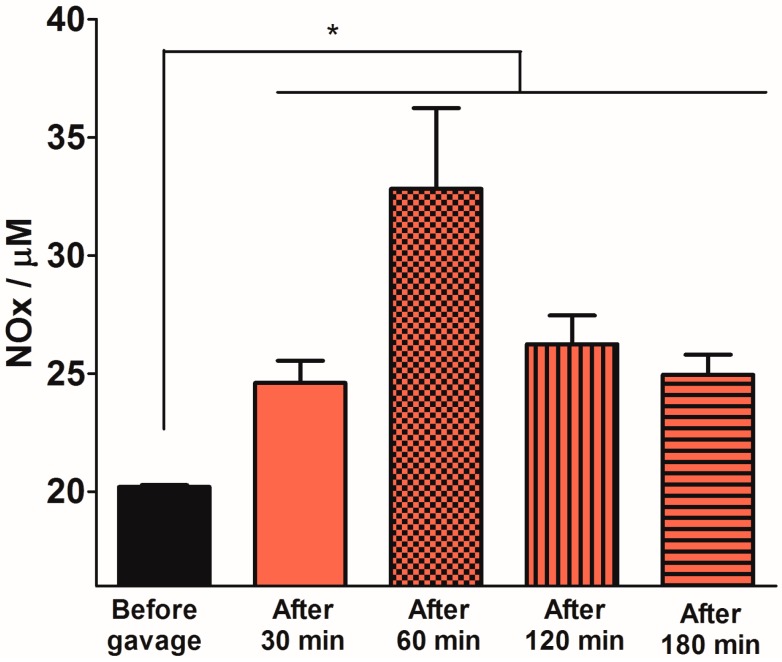
Plasma NOx levels 30, 60, 120 and 180 min after 12 mM oral SNAC administration to Swiss mice. The results are expressed as the mean ± S.E.M. (*n* = 3). * *p* < 0.05 compared to “Before gavage” group, one-way ANOVA with Bonferroni *post hoc* test.

**Figure 6:**
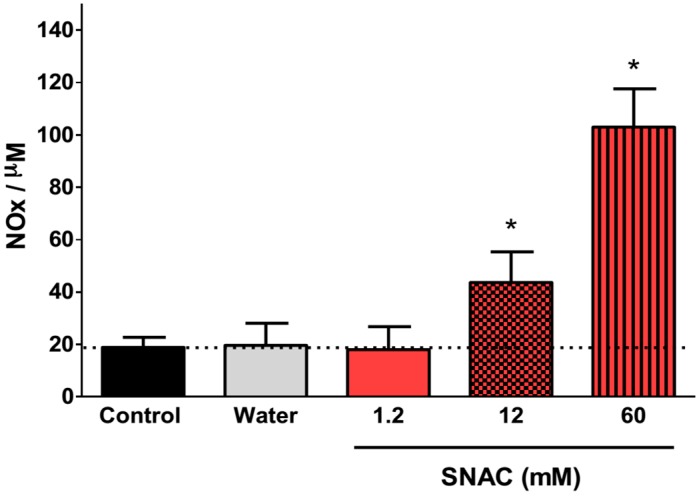
Dose-response of plasma NOx concentration 60 min after 1.2, 12 and 60 mM oral SNAC administration. The results are expressed as the mean ± S.E.M. (*n* = 3). * *p* < 0.05 compared to control animals, one-way ANOVA with Bonferroni *post hoc* test.

Figure 5 shows the time-course of the plasma NOx concentrations 30, 60, 120 and 180 min after oral 12 mM SNAC administration to Swiss mice. We observed a significant increase in absolute plasma NOx levels at all time points, and the maximum increase (relative to the basal level) occurred after 60 min. After this time, plasma NOx levels began to decrease, although the levels were still significantly higher than basal levels 2 h after gavage. Figure 6 shows the dose-response of plasma NOx concentration 1 h after 1.2, 12 and 60 mM (corresponding to 7.0 μmol/kg, 70 μmol/kg and 350 μmol/kg, respectively) oral SNAC administration to Swiss mice compared to control naive animals and to animals that received only distilled water. While the plasma NOx concentration of the animals that received 1.2 mM SNAC did not significantly change compared to control animals (approximately 20 μM), 12 and 60 mM SNAC administration led to a significant dose-response increase in plasma NOx concentrations, which reached 45 ± 12 µM and 103 ± 15 µM, respectively. For this reason, we chose 12 mM SNAC and equimolar NaNO_2_ and NaNO_3_ solutions as the highest concentrations for evaluating their effects on the GMBF increase and time course of plasma NOx levels after oral SNAC administration, as described above.

### 2.4. Discussion

**Scheme 1:**
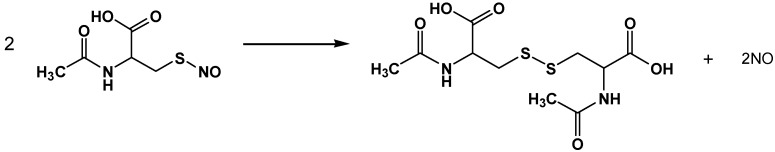
Spontaneous NO release from SNAC.

This is the first study to our knowledge where an *ex vivo* gastric chamber method was used to investigate the effect of luminal SNAC administration on GMBF and where the physiological effects of SNAC were correlated with the rates of spontaneous NO release from SNAC solutions. Characterization of these rates showed that aqueous SNAC solutions continuously release free NO after its formation from NAC S-nitrosation. The initial narrow peaks observed in chemiluminescence NO detection (Figure 2) can be assigned to spontaneously released NO by SNAC molecules, which accumulates in the solutions prior to analysis. Immediately after solution injection into the NO analyzer reaction flask, which is continuously flushed by N_2,_ the accumulated NO is immediately carried to the detector, resulting in the observed narrow transient peaks. We confirmed the rapid free NO accumulation in SNAC solutions by allowing NO to re-accumulate in the solution after its removal by the instrument’s N_2_ flow, (Figure S3), as interruption of N_2_ flow after recording the first narrow peak leads to new narrow peaks upon N_2_ flow reestablishment. The plateau signal that follows the initial NO peak reflects steady NO production from SNAC during the measurement time period. NO generated in both the peak and the plateau comes from the bimolecular reaction of two SNAC molecules to form a sulfur-bridged NAC dimer with the concomitant release of two NO molecules according to Scheme 1.

The rate of reaction 1 depends on SNAC concentration, as previously reported [17]. Thus, the rapid GMBF increase immediately after luminal SNAC application on the gastric mucosa can be assigned to the NO released in reaction 1. Increased GMBF maintenance while the gastric mucosa is incubated with the SNAC solutions is likely due to the approximately constant rate of NO released during the plateau phase of SNAC decomposition, as shown in the kinetic curves of Figure 2A,B. NO released in scheme 1 is expected to diffuse through the mucous layer of the gastric mucosa, the mucosal capillary bed and the muscularis mucosa, reaching the arterial and venous plexuses of the submucosa. Through this diffusion process, NO can activate soluble guanylate cyclase (sGC) in the smooth muscle cells (SMC) of the mucosal capillary bed and of the arterial and venous plexuses of the submucosa microvasculature, including the arterioles and the capillary network, which drains into the venules that accompany the arterioles. sGC activation in the microvasculature SMCs elicits a cyclic guanosine monophosphate (cGMP)-dependent vasodilatory response, according to the well-known NO-mediated vasodilation mechanism [18].

The observed GMBF increase can be assigned exclusively to exogenous NO released by SNAC and is independent of the endothelium, which is itself a major source of NO and of other vasoactive paracrine factors. Although the NO release rate from the 12 mM SNAC solution (14 nmol·mL^−1^·min^−1^) is 4.7 times higher than from 600 μM SNAC (0.3 nmol·mL^−1^·min^−1^), there was no significant difference between the maxima GMBF plateaus from these two solutions. These results suggest that the maximum subepithelial microcirculation vasodilation was already achieved with 600 μM SNAC. The apparent microvasculature tolerance to further increases in blood flow with higher SNAC concentrations is likely associated with the maximum mechanically possible SMC relaxation. However, other mechanisms, such as the depletion of endogenous species involved in cGMP-dependent NO signaling to vasodilation or to metabolic and myogenic mechanisms that operate in the auto-regulatory responses in arteries and arterioles to restore blood flow to basal conditions, cannot be ruled out.

**Scheme 2:**
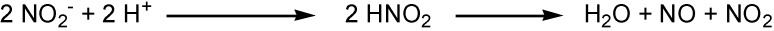
Nitric oxide release from acid nitrite solution.

The increased GMBF upon 600 μM SNAC application has great potential to counteract NSAID-induced injuries, at least in in the present animal model, considering that a maximum GMBF decrease of 25% was observed in indomethacin or diclofenac-treated Sprague Dawley rats [19], while in the present study, luminal SNAC application resulted in GMBF increases greater than 250% in the same animal model. In contrast, NaNO_2_ and NaNO_3_ solutions did not affect GMBF in the conditions used in the present study. Although Petersson *et al.* [20] reported a similar study in which NaNO_2_ incubation increased GMBF, the authors used acidic NaNO_2_ solutions in which NaNO_2_ is converted to nitrous acid (HONO), which undergoes decomposition to release free NO (Scheme 2).

This is also the fate of nitrite coming from the reduction of dietary nitrate, which is known to be metabolized *in vivo* to NO, a pathway involved in the mediation of blood flow regulation [21]. Therefore, in the normal situation the administration of nitrite to healthy patients with acidic luminal pH, is expected to lead to NO production with consequent GMBF increase. In the *ex vivo* gastric chamber condition of the present study the normal protective effect of the buffered stomach mucous layer can be compromised, thus the SNAC, NaNO_2_ and NaNO_3_ solutions were buffered at pH 7.4 in order to avoid any possible artifact due to gastric acid damage caused by the administration of these solutions at the acidic luminal pH 1–2. A special point must be raised for patients receiving proton pump inhibitor (PPI) co-therapy with NSAIDs. In these cases, the intragastric pH can be higher than 6 [22,23] and the administration of nitrite or nitrate to increase GMBF would be ineffective, while SNAC administration could enhance the protective action of the PPI therapy. In this respect, the characterization of the effects of SNAC, NaNO_2_ and NaNO_3_ on the GMBF at pH 7.4 allows proposing a potential therapeutic action of SNAC for patients taking PPI. In addition, as nitrate is incapable of generating NO in the absence of nitrate reductases, luminal administration of NaNO_3_ solution at pH 7.4 was used as a negative control in our study.

Our chemiluminescence measurements of NO release from 12 mM NaNO_2_ solution (pH 7.4) showed a rate of NO formation of 0.06 nmol·mL^−1^·min^−1^, which is fivefold lower than the rate of NO released from 600 μM SNAC, and this does not lead to significant blood flow increase as measured by Laser Doppler Flowmetry. In the case of 12 mM NaNO_3_ solution, no equilibrium implying NO production can be established in the absence of NO_3_^−^ to NO_2_^−^ reducing agents, which in turn, could produce NO according to reaction 2. This hypothesis is supported by the absence of vasodilation upon 12 mM NaNO_3_ application (Figure 2D) and the absence of chemiluminescence of NO release. In this case, the quantitatively insignificant narrow NO peak (Figure 2D), can be assigned to the presence of trace amounts of NaNO_2_ in the NaNO_3_ reagent. We also investigated the kinetic behavior of NO release from SNAC solutions 600 μM and 12 mM at the pH 1.2 using a simulated gastric fluid (SGF) medium. The results obtained are shown in Figure S4 where it can be seen that in the SGF both the NO release profile and the rate of NO release from the SNAC solution 600 μM are very similar to those obtained at pH 7.4. However, the SNAC solution 12 mM showed a different NO release profile, which led to a much higher rate of NO release. The similar behavior of the SNAC 600 μM solution at pH 7.4 and 1.2 is in accordance with previous studies which showed that GSNO, also a primary S-nitrosothiol, has an enhanced stability in highly acidic pH [24]. The higher rate of NO release observed for the SNAC solution 12 mM at pH 1.2 may be associated with the catalytic action of trace metal ions present in the SGF reagents, and is expected to be more prominent in concentrated S-nitrosothiol solutions, which are subjected to autocatalytic effect on their thermal decomposition [17]. For therapeutic purposes, one may consider that the less concentrated SNAC solution 600 uM is already capable of promoting the maximum GMBF increase, as shown in Figure 4, and that its rate of NO release will not be significantly affected by the stomach pH in the range 1.2 to 7.4.

The profile of plasma NOx levels after SNAC administration shows a significant increase after 30 min reaching a maximum after 60 min, compared to the basal level. This profile suggests that, despite the fast NO release from the SNAC solutions displayed in Figure 2, the increase in plasma NOx concentration is governed by the kinetics of NOx absorption in the gastric mucosa, followed by the distribution of these species in the systemic circulation. , The decrease of the plasma NOX levels after 1 h, reflects the fast diffusion of the primary NO products (NO_2_^−^ and NO_3_^−^) though the stomach and intestine and a fast clearance of these anions from the blood. Our results are in accordance with those reported by Pannala *et al.* who found that urinary excretion in humans leads to total nitrate clearance in a 24-h period [25]. In addition, the basal NOx levels (approximately 20 μM) are also in accordance with previous reports of plasma and urinary NOx levels in animals. For example, Fletcher *et al.* [26] reported plasma NOx levels of approximately 17 μM in naive Lewis rats, compared to values up to 56 ± 18 μM under inflammatory conditions after arthritis induction. Amsterdam *et al.* [27] analyzed Wistar rats and rabbits and found basal plasma NOx levels of 34 and 61 μM, respectively. Plasma NOx measurements in diabetic rats also led to values in the same order of magnitude, such as those reported by Kino *et al.* [28] of 20 μM for control Wistar rats and 12 and 14 μM for diabetic Wistar rats, a condition of impaired NO production.

Notably, only oral 12 and 60 mM SNAC administration (corresponding to 70 μmol/kg and 350 μmol/kg, respectively) led to significant increases in average plasma NOx levels in mice. However, luminal 600 μM SNAC administration (a concentration 20 times lower than 12 mM) on rat stomachs was sufficient for a marked increase in GMBF. Therefore, the local actions of SNAC, such as GMBF increase, do not necessarily imply increases in plasma NOx above basal levels. Similarly, other studies have reported therapeutic and protective actions of SNAC using much lower doses and concentrations, such as the attenuation of liver fibrosis in cirrhotic rats with 6 μmol/kg/day oral SNAC [16], and the reduction of ischemia reperfusion lesions in the steatotic liver [14] and a protection of livers during cold storage [29] in rats, both using 0.1 μM SNAC. Therefore, the potential therapeutic actions of SNAC may be obtained in an ample concentration range without necessarily impacting the homeostatic plasma NOx levels.

**Figure 7:**
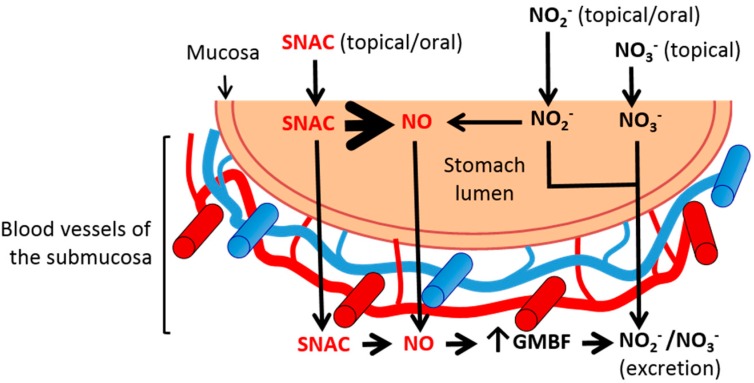
Scheme of the major pathways involved in the vasodilation action of SNAC on the gastric mucosal blood flow (GMBF) compared to nitrite (NO_2_^−^) and nitrate (NO_3_^−^) anions. Orally or luminally administered SNAC spontaneously releases NO into the stomach lumen. Free NO diffuses through the gastric mucosa into the submucosal blood vessels, where it increases GMBF. Intact SNAC molecules may also diffuse through the gastric mucosa into the submucosal blood vessels, where *in situ* NO release may lead to vasodilation. At neutral pH, NO_2_^−^ and NO_3_^−^ anions from oral or luminal sources diffuse to the submucosal blood vessels where they contribute to NO-derived NO_2_^−^ and NO_3_^−^ levels and are excreted without increasing GMBF. The blood vessels of the submucosa are not drawn to scale. SNAC, S-nitroso-N-acetylcysteine; NO, nitric oxide.

Finally, further studies are necessary to investigate whether SNAC completely decomposes in the stomach lumen after administration, and only the free NO diffuses through the gastric mucosa leading to GMBF increase with a subsequent plasma NOx increase, or whether intact SNAC also crosses the gastrointestinal barrier into the submucosal microcirculation, where it may release NO *in situ*, also leading to GMBF increase. In any case, at a neutral pH, the contribution of exogenous NO_2_^−^ to free NO production is negligible, and both NO_2_^−^ and NO_3_^−^ ions likely follow excretion pathways without increasing GMBF. These mechanistic possibilities are schematically shown in Figure 7.

## 3. Experimental Section

### 3.1. SNAC Synthesis

S-nitroso-N-acetylcysteine (2-acetamido-3-nitrososulfanylpropanoic acid, C_5_H_8_N_2_O_4_S, MW: 192.193020 g/mol) was synthesized from equimolar amounts of aqueous NaNO_2_ and NAC at pH 2, which was adjusted with 6 M HCl. The reaction was performed at room temperature with constant stirring in an ice bath for 20 min. After the reaction was complete, the acidic solution was neutralized to pH 7.0 with NaOH. The final 600 μM and 12 mM SNAC solutions were used immediately. SNAC solutions were spectrophotometrically characterized by obtaining their UV-Vis spectra (200–800 nm) in the concentration range of 10 to 1000 μM in a quartz cuvette (1 cm optical path) at 25 °C using a model 8453 spectrophotometer (Hewlett-Packard, Santa Clara, CA, USA) equipped with a Peltier thermostated cell holder (Figure S1).

### 3.2. Characterization of NO Release from SNAC, NaNO_2_ and NaNO_3_ Solutions

The spontaneous NO release from 600 μM and 12 mM SNAC solutions in PBS (pH 7.4) was characterized by chemiluminescence using a Nitric Oxide Analyzer (NOA 280i, GE Analytical Instruments, Boulder, CO, USA) operating at 6.1 psi O_2_ pressure and 6.5 Torr N_2_ pressure [30]. A total of 5 mL PBS was initially placed in the instrument’s reaction flask and thermostated at 37 °C. After baseline stabilization, 600 μM and 12 mM SNAC solutions and 12 mM NaNO_2_ and NaNO_3_ solutions were analyzed by injecting 100 µL of the individual SNAC, NaNO_2_ or NaNO_3_ solutions and by monitoring the NO release response over 10 min. The instrument was initially calibrated with standard NaNO_2_ solutions as described previously [31] (Figure S2). Measurements were performed in triplicate and are expressed as the mean ± SD.

### 3.3. Animal Care

The present study was performed in accordance with the guidelines of standard humane animal care as outlined in the “Guide for the Care and Use of Laboratory Animals”, National Academy Press, 1996. The Ethical Committee of the University of Campinas approved all animal procedures. The study was performed using healthy male Sprague Dawley rats (*n* = 3) weighing between 250 and 300 g and healthy male Swiss mice weighing between 30 and 35 g (*n* = 4–5). All animals were provided by the Multidisciplinary Center for Biological Research in Laboratory Animals of the State University of Campinas (CEMIB/Unicamp). All animals were housed in ventilated clean cages (dimensions 30.0 × 20.0 × 13.0 cm (mice) or 49.0 × 34.0 × 16.0 cm (rats)) in standard housing conditions (12 h light and 12 h darkness, 25 °C) which were cleaned twice a week. All animals had free access to food and water.

### 3.4. Experimental Design

The effects of local SNAC, NaNO_2_ and NaNO_3_ solution administration on GMBF were measured using the *ex vivo* gastric chamber method described by Camara *et al.* [32]. A pencil probe (type N, penetration 1 mm, Transonic Systems, Ithaca, NY, USA) connected to a Laser Doppler Flowmeter (BLF 21A, Transonic Systems) was used in all cases. Male Sprague Dawley rats (*n* = 3), which had been deprived of food but not water for the previous 6 h, were used. The animals were anesthetized with halothane and laid supine in a Plexiglas holder heated from an underneath heating pad at 37 °C. Their abdomens were opened by midline incision, and the stomach was exposed and carefully opened along the greater curvature. The gastric mucosa was exposed, everted onto a 1.5-cm-diameter hole in a round Plexiglas plate and pinned along the hole’s perimeter. The pinned gastric mucosal border was further clamped with a Plexiglas cylinder, which served as a chamber to apply the solutions to the exposed stomach lumen. The gastric mucosa was washed three times with 5 mL PBS (pH 7.4) and then incubated in 5 mL PBS (pH 7.4) for three min to record the basal GMBF. The PBS was then replaced by fresh PBS (5 mL) in the control animals or by 5 mL aqueous SNAC (600 μM or 12 mM), NaNO_2_ (12 mM) or NaNO_3_ (12 mM) solution in the treated animals, and the GMBF was monitored for 10 min. The mucosa was washed again three times with normal PBS, and the SNAC solution was replaced by 5 mL PBS (pH 7.4). The GMBF was measured for an additional 7 min. The GMBF values were recorded every minute and expressed as perfusion units normalized to control values, which were obtained from the PBS bath. Three animals were studied in each group. The animals were killed under deep anesthesia by decapitation.

The time and dose-responses of oral gavage SNAC administration on total plasma NO metabolites were assessed by quantifying the sum of NO_2_^−^ and NO_3_^−^ levels; the sum was termed NOx. Swiss mice were used for gavage experiments instead of Sprague-Dawley rats because they are better suited for repeated tail blood collection through tail vein prick with minimal animal restraint and stress, while tail blood collection from rats would demand tail tip removal under terminal anesthesia. Swiss male mice (*n* = 4–5) were fasted for 6 h with free access to distilled water and evaluated in gavage experiments.

To evaluate the time-response of plasma NOx levels, the animals were fasted and gavaged with 200 μL 12 mM SNAC. Blood samples were collected after 30, 60, 120 and 180 min. To evaluate the dose-response of plasma NOx levels, the animals were fasted and gavaged with 200 μL distilled water (control group) or 200 μL SNAC solutions at three different concentrations: 1.2, 12 and 60 mM, which corresponded to doses of 7, 70 and 350 μmol/kg, respectively. Tail blood was collected after 1 h, at which time the maximum NOx level was observed, using a non-surgical microsampling technique. Each mouse was removed from the home cage, placed on top of the bench, gently restrained by the base of the tail, and the tip of the tail was pricked with a sterile 23–25-gauge needle. The blood sample was collected by capillary action using a 10 μL microcapillary tube inserted into a pipette bulb, which was placed near the blood sample at the tip of the tail. Blood flow was stopped by applying finger pressure to the soft tissue for approximately 20 s before returning the mouse to its home cage. The blood samples were dispensed into heparin-coated tubes and centrifuged at 3000 rpm for 30 min at room temperature. Plasma NOx levels were measured by chemiluminescence. Briefly, measurements were performed by the vanadium chloride method to ensure the complete reduction of NO_2_^−^ and NO_3_^−^ anions to NO [33]. The vanadium chloride solution (6 mL) was initially placed in the instrument’s reaction flask and thermostated at 90 °C. After baseline stabilization, 1 μL of the plasma sample was injected, and the NO release signal was monitored. In these measurements, O_2_ and N_2_ pressures were set at 6.0 psi and 7.0 Torr, respectively. A calibration curve was first obtained from injections (1 µL) of five standard NaNO_3_ solutions in the concentration range of 1 to 1,000 µM. Measurements of each sample were performed in triplicate.

### 3.5. Materials

N-acetylcysteine (NAC), sodium nitrite (NaNO_2_), sodium nitrate (NaNO_3_), phosphate buffer saline (PBS), vanadium chloride, sodium iodide (KI), glacial acetic acid and hydrochloric acid were purchased from Sigma (St. Louis, MO, USA). Deionized water from a Milli-Q purification system was used to prepare all solutions.

### 3.6. Statistical Analysis

Results are expressed as mean ± S.E.M. Statistical differences between the groups were analyzed using one-way ANOVA followed by Bonferroni *post hoc* test. A difference with a *p* < 0.05 was considered statistically significant.

## 4. Conclusions

Luminal 600 μM and 12 mM SNAC administration, but not 12 mM NaNO_2_ or NaNO_3_ solutions, led to a rapid threefold increase in the GMBF of rats, which was maintained throughout the incubation with these solutions. This effect correlated to the sixfold higher rate of spontaneous NO release from SNAC solutions, compared with equimolar NaNO_2_ and is in accordance with the absence of NO release from the NaNO_3_ solution. Oral SNAC administration led to a dose-response increase in plasma NOx levels compatible with physiological action based on NO release from SNAC. Oral SNAC administration may provide a new therapeutic approach for acid-peptic disorder prevention and treatment.

## Supplementary Materials

Supplementary materials can be accessed at: http://www.mdpi.com/1420-3049/20/03/4109/s1.
