# Supplementary material for: Nitric Oxide Released from Luminal S-Nitroso-N-Acetylcysteine Increases Gastric Mucosal Blood Flow
Source: Molecules. 2015 Mar 4;20(3):4109–23. doi: 10.3390/molecules20034109 (PMC6272716; doi:10.3390/molecules20034109)
Supplement: Supplementary file 1 [file molecules-20-04109-s001.pdf]

Supplementary Materials

S1. Spectrophotometric Characterization of the SNAC Solutions

Figure S1 shows the spectra of aqueous SNAC solutions in the range of 100 to 1000 µM. The spectra display the characteristic intense band of primary S-nitrosothiols with maximum at 336 nm, assigned to the π-π^* electronic transition of the SNO group. The spectrum of the 1000 µM solution also displays the much weaker absorption band of SNAC at 545 nm, assigned to the n-π^* electronic transition of the SNO group [1]. The linearity obtained in the calibration curve show that the 336 band is adequate for the spectrophotometric monitoring and quantification of aqueous SNAC solutions in the range of 100 to 1000 µM, while above 1000 µM, the 545 nm band can be used for this purpose. The molar absorption coefficient calculated from the linear regression of the data in Figure S1B is $\varepsilon = 898\ M^{-1}\cdot cm^{-1}$, what is similar to values previously reported for SNAC and GSNO [1,2] which are within the range of 836–922 $M^{-1}\cdot cm^{-1}$.

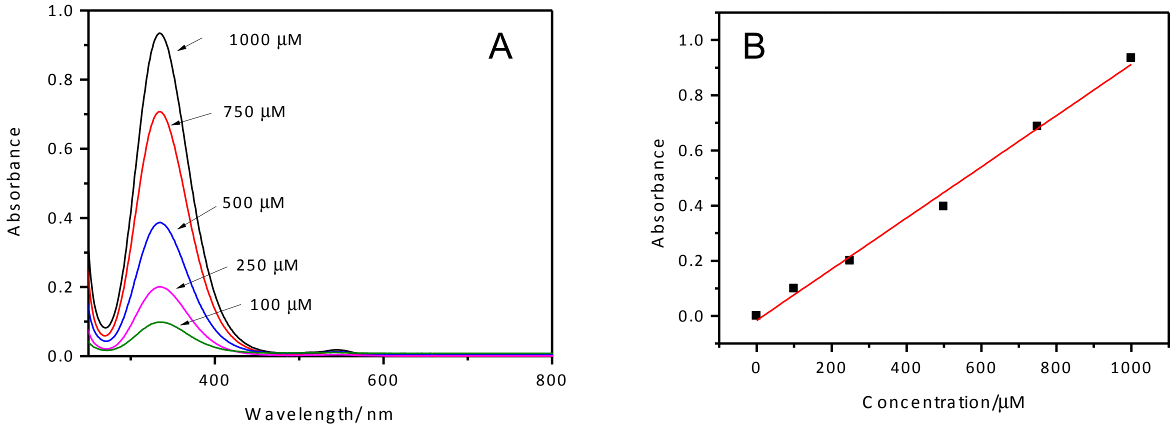

Figure S1. (**A**) UV-Vis spectra of freshly prepared S-nitroso-N-acetylcysteine (SNAC) solutions at the concentrations 0 (Control = background spectrum of water), 100, 250, 500, 750 and 1000 µM. (**B**) Corresponding calibration curve for the SNAC solutions of (A). Correlation coefficient = 0.9921; standard deviation = 0.010.

S2. Calibration of the Chemiluminescence NO Analyzer

The calibration of the Nitric Oxide Analyzer (NOA 280i, GE Analytical instruments, Boulder, CO, USA) was performed using standard $NaNO_2$ solutions, based on the quantitative NO release from $NaNO_2$ due to its reduction by KI in the presence of acetic acid. Calibration curves obtained in the high and low sensitivity modes of the instrument are shown in Figure S2.

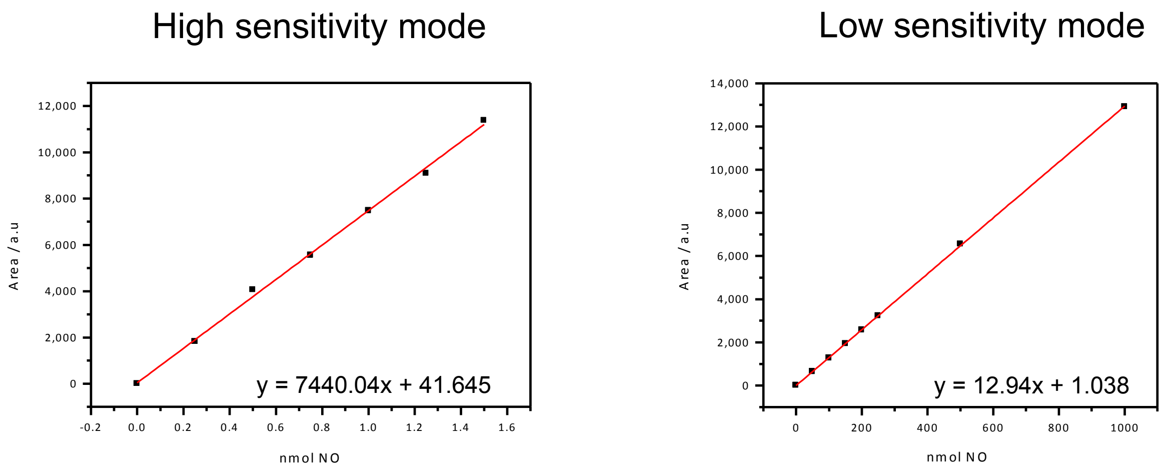

Figure S2. Calibration curves obtained for the monitoring of nitric oxide released from SNAC, sodium nitrite and sodium nitrate solutions by chemiluminescence, in the high sensitivity and low sensitivity modes.

S3. Chemiluminescence Detection of NO Released from SNAC Solutions 12 mM

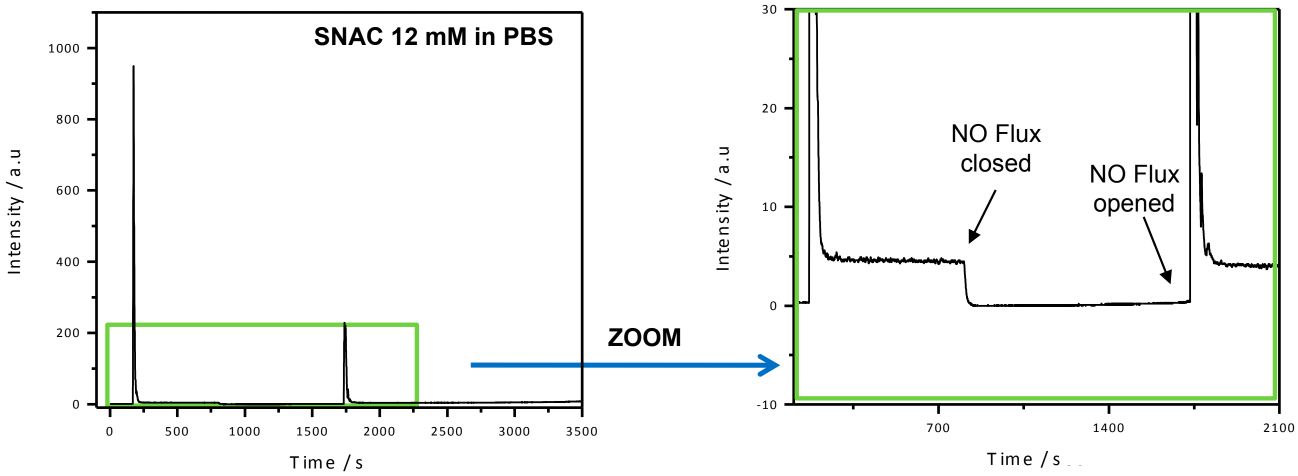

Figure S3. Chemiluminescence detections of NO released from SNAC solutions 12 mM at 37 °C under constant removal by the N_2 flow of the instrument. Interruption of the N_2 flow after recording the first narrow NO peak, and reestablishment of the N_2 flow at the time 1750 s, leads to the detection of a new narrow NO peak. The magnified figure on the right, shows that quitting N_2 flow also brings the plateau back to the base line, as expected, confirming that the plateau is due to a constant NO release from the SNAC solution.

S4. Chemiluminescence Detection of NO Released from SNAC Solutions 600 µM and 12 mM in Simulated Gastric Fluid (SGF).

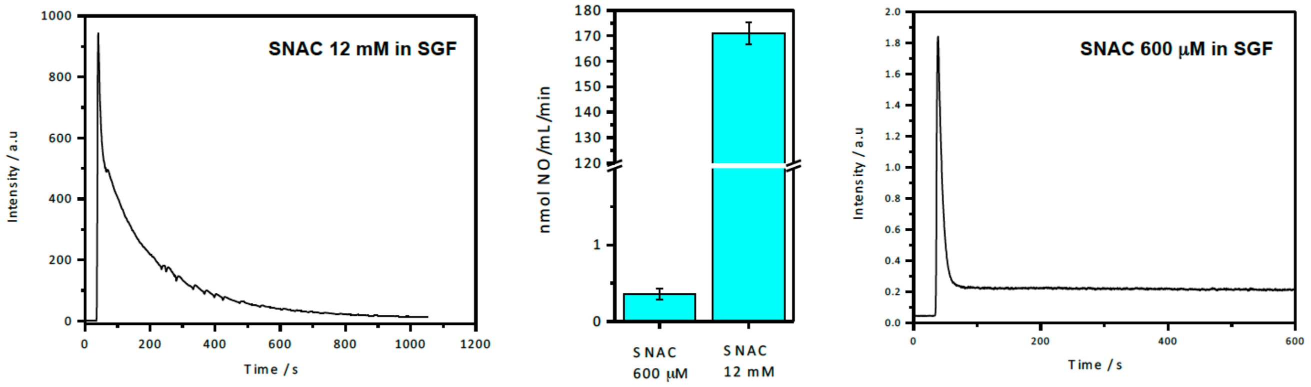

Figure S4. Chemiluminescence detections of NO released from SNAC solutions 600 µM and 12 mM in simulated gastric fluid, pH 1.2 at 37 °C. A total of 5 mL SGF was initially placed in the instrument's reaction flask and thermostated at 37 °C. After baseline stabilization, 600 µM and 12 mM SNAC solutions were analyzed by injecting 100 µL of the individual SNAC, solutions and by monitoring the NO release response. The instrument was initially calibrated with standard $NaNO_2$ solutions as described previously. SGF, pH 1.2, was prepared by dissolving 2 g of sodium chloride in 7 mL HCl and sufficient water to make 1 L, according to ref. [3].

References

1. De Oliveira, M.G.; Shishido, S.M.; Seabra, A.B.; Morgon, N.H. Thermal stability of primary s-nitrosothiols: Roles of autocatalysis and structural effects on the rate of nitric oxide release. *J. Phys. Chem. A* **2002**, *106*, 8963–8970.
2. Katarzyna, A.; Broniowska, K.A.; Diers, A.R.; Hogg, N. S-nitrosoglutathione. *Biochim. Biophys. Acta* **2013**, *1830*, 3173–3181.
3. Singh, S.; Mariappan, T.T.; Sharda, M.; Singh, B. Degradation of Rifampicin, Isoniazid and Pyrazinamide from Prepared Mixtures and Marketed Single and Combination Products under Acid Conditions. *Pharm. Pharmacol. Commun.* **2000**, *6*, 49–494.
